# Computational analysis of amino acids and their sidechain analogs in crowded solutions of RNA nucleobases with implications for the mRNA–protein complementarity hypothesis

**DOI:** 10.1093/nar/gku1035

**Published:** 2014-10-31

**Authors:** Matea Hajnic, Juan Iregui Osorio, Bojan Zagrovic

**Affiliations:** Department of Structural and Computational Biology, Max F. Perutz Laboratories, University of Vienna, Vienna 1030, Austria

## Abstract

Many critical processes in the cell involve direct binding between RNAs and proteins, making it imperative to fully understand the physicochemical principles behind such interactions at the atomistic level. Here, we use molecular dynamics simulations and 15 μs of sampling to study the behavior of amino acids and amino acid sidechain analogs in high-concentration aqueous solutions of standard RNA nucleobases. Structural and energetic analysis of simulated systems allows us to derive interaction propensity scales for different amino acid/nucleobase combinations. The derived scales closely match and greatly extend the available experimental data, providing a comprehensive foundation for studying RNA–protein interactions in different contexts. By using these scales, we demonstrate a statistically significant connection between nucleobase composition of human mRNA coding sequences and nucleobase interaction propensities of their cognate protein sequences. For example, pyrimidine density profiles of mRNAs match uracil-propensity profiles of their cognate proteins with a median Pearson correlation coefficient of *R* = −0.70. Our results provide support for the recently proposed hypotheses that mRNAs and their cognate proteins may be physicochemically complementary to each other and bind, especially if unstructured, with the complementarity level being negatively influenced by mRNA adenine content. Finally, we utilize the derived scales to refine the complementarity hypothesis and closely examine its physicochemical underpinnings.

## INTRODUCTION

From transcriptional and translational regulation to RNA processing and decay to protein localization, many key processes in the cell depend directly on RNA–protein interactions (1–4). What is more, the list of systems that involve RNA–protein interactions keeps dramatically expanding. Recently, for example, high-throughput efforts aimed at capturing the mRNA–protein interactome identified a large number of novel RNA-binding proteins (5,6). Out of a total of approximately 800 mRNA-binding proteins detected in these studies using covalent UV-crosslinking methods, about 25% were found not to contain any known RNA-binding domains, while an even greater number lacked clear functional characterization. Despite the challenges ahead, one may expect that integrative efforts involving biochemical, structural and computational techniques will soon catalog most if not all of biologically relevant RNA–protein interactions. On the other hand, our understanding of the basic physicochemical principles behind such interactions still remains incomplete. Most importantly, only a few experimental studies have been performed in order to directly explore interactions between individual nucleobases and amino acids in different environments (7–10). While global and local structural contexts do play important roles in defining the properties of RNA–protein binding interfaces, it is reasonable to expect that binding specificity in general also critically depends on the preferences of individual nucleobases and amino acids for each other.

In this reductionist framework, the properties of the binding sites are at least in part a consequence of binding preferences that are intrinsic to individual nucleobases and amino acids. Motivated by this, Akinrimisi *et al.* (9) and Thomas *et al.* (10) have measured affinities of several naturally occurring amino acids for a set of nitrogenous bases and nucleosides using spectroscopic methods, but those experiments were never performed systematically for all possible combinations. Furthermore, Woese *et al.* have used chromatographic measurements to define a scale of amino acids’ propensity to interact with pyrimidine mimetics pyridines, which they termed ‘polar requirement’ (PR) (7,8). Finally, several authors have studied interactions between different nucleotides and polyamino acids, focusing typically on polylysine or polyarginine peptides (11–13). Despite the clear importance of such experimental studies, however, we find it remarkable that they have not been repeated or extended since the 1960s and 1970s when they were first performed. On the other hand, sizable progress has been made using computational and theoretical approaches (14–28). Most significantly, the available structures of nucleic acid–protein complexes have been statistically analyzed to explore the more general physicochemical principles behind nucleobase/amino acid interactions (14–18) and, in particular, derive binding preference scales, also known as knowledge-based potentials (19–23). Moreover, a computational equivalent of the PR scale was derived using molecular dynamics (MD) simulations, providing a microscopic picture behind the interaction propensities exhibited by individual amino acids (24). Finally, quantum-mechanical calculations have been used to characterize the interactions between a select subset of bases and amino acids (25–28). Overall, all of these studies suggest that the preferences of individual nucleobases and amino acids for each other in water may be highly differentiated, but a large-scale analysis of this effect has never been systematically performed.

An important context in which nucleobase/amino acid interactions may be relevant concerns an important foundational question in molecular biology, that of the origin of the universal genetic code (29–31). In particular, the stereochemical hypothesis proposes that the code evolved as a consequence of direct interactions between codons and amino acids they code for (7,8,32–35). An early formulation of the stereochemical hypothesis was put forth by Carl Woese *et al.* based on the above mentioned PR scale, i.e. the propensity of amino acids to interact with pyrimidine mimetics (7,8). Recently, we have demonstrated that pyrimidine density profiles of mRNA coding sequences closely mirror the PR-weighted profiles of their cognate protein sequences (36). In other words, pyrimidine-rich mRNA regions tend to code for cognate protein regions that exhibit high propensity to interact with pyrimidine mimetics and vice versa. Moreover, we have used knowledge-based potentials derived from experimental structures of RNA–protein complexes to not only confirm these findings in the case of pyrimidines, but also extend them to purines (23,37). By providing quantitative evidence for an early, more qualitative proposal by Kyrpides and Ouzounis (38,39), these results allowed us to raise the stereochemical hypothesis to the level of a general relationship between sequence composition of mRNAs and their cognate proteins as well as to hypothesize that mRNAs could bind their cognate proteins in a complementary fashion, especially if unstructured (23,36,37). We argued that such binding interactions were an important driving force behind the establishment of the universal genetic code, but also that they may have a critical, yet still not fully characterized role in present-day cell as well (23,36,37). Intriguingly, in our analysis of knowledge-based preference scales, we found that guanine and adenine exhibit opposite amino acid binding preferences, resulting in a curious asymmetry: while guanine-binding propensity profiles on the side of proteins closely match purine density profiles on the side of their cognate mRNAs, adenine-binding protein sequence profiles more closely mirror pyrimidine density mRNA profiles (23,37). This hints at additional complexities behind the putative cognate complementarity and suggests that there may have been at least two major phases in the development of the genetic code with opposite requirements when it comes to complementary matching (37).

**Figure 1.**
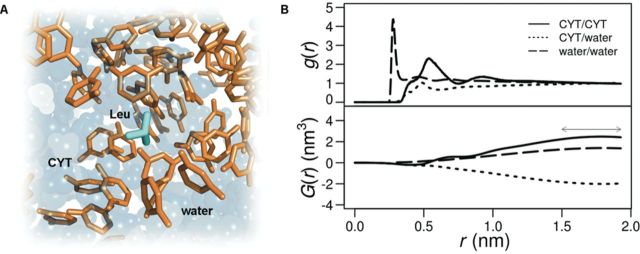
(**A**) A typical snapshot from the simulation with a single amino acid sidechain analog (Leu) in CYT/water mixture. (**B**) CYT/CYT, CYT/water and water/water radial distribution functions in Leu simulations (top) with the corresponding Kirkwood–Buff integrals (bottom). The final value for the integrals was taken as the average over the approximately constant window denoted by the arrow.

In order to better understand the underlying physicochemical principles behind RNA–protein interactions and shed more light on the mRNA/protein complementarity hypothesis, here we systematically explore the behavior of individual amino acids and amino acid sidechain analogs in high-concentration aqueous solutions of different RNA nucleobases using classical MD simulations (Figure 1A). For our simulations, we employ the GROMOS 53A6 force field (40), which was parameterized to accurately capture solvation free energies of amino acid sidechain analogs in water and cyclohexane. These hydrophobicity-related properties, in turn, are considered to be an important factor in defining amino acid/nucleobase interaction propensities. Empowered by the spatial and temporal resolution provided by MD simulations, we present an atomistic view of how individual amino acids or sidechain analogs interact with RNA nucleobases in water and define interaction preferences between them using different structural and energetic criteria. Such interaction propensity scales create a rigorous, reductionist foundation for the analysis of RNA–protein interactions in general, which is here further employed for a critical examination of the mRNA–protein complementarity hypothesis and its refinement.

## MATERIALS AND METHODS

The 20 natural amino acids and 18 of their sidechain analogs (all except for Gly and Pro) were simulated in the presence of a single type of common RNA nucleobases in aqueous solution: adenine (ADE), cytosine (CYT), guanine (GUA) or uracil (URA). In all simulations, a single amino acid or a sidechain analog (corresponding to an amino acid residue with a hydrogen atom added to Cβ) was centered in a cubic box of initial size 4 × 4 × 4 nm^3^ with nucleobases and water molecules placed in random orientations around it so as to achieve the molar fraction of water of 0.86. Considering the low water solubility of naturally occurring nucleobases, this molar fraction of water was chosen in order to reach a compromise between maximizing the probability of detecting interaction between amino acids (i.e. their sidechain analogs) and nucleobases on the one hand and minimizing nucleobase solubility issues on the other. In total, there were approximately 1250 molecules in each system: one amino acid or sidechain analog, 170 nucleobase molecules and the rest water molecules (see Supplementary Table S1 for details). All amino acids were simulated in their zwitterionic form. In the case of charged amino acids or sidechain analogs, one randomly chosen water molecule was replaced by a counter ion (Na^+^ or Cl^−^) in order to obtain an electrically neutral system.

All simulations were carried out using the Gromacs 4.5.1. simulation package (41), united-atom GROMOS 53A6 force field (40) and SPC/E water model (42) with a 2 fs integration step. Parameters for the nucleobases were obtained from those corresponding to full nucleotides in the GROMOS 53A6 force field while ensuring charge neutrality. Long-range electrostatic interactions were treated using Particle Mesh Ewald (PME) summation with a grid spacing of 0.12 nm and an interpolation order of 4. The cut-off for short-range Coulombic and van der Waals interactions was set to 0.9 nm. The temperature and pressure in all simulations were kept at 300 K and 1 bar using V-rescale thermostat (τ_T_ = 0.1 ps) (43) and Parrinello-Rahman barostat (τ_p_ = 2 ps and compressibility = 4.5 × 10 ^−5^ bar ^−1^) (44), respectively. After minimization using the steepest descent algorithm in water (10 000–25 000 steps), the systems were first equilibrated in the NVT ensemble for 800 ps and then subjected to 400 ps of equilibration in the NPT ensemble with the same position restraints placed on the amino acid or sidechain analog. All production runs, each 100 ns long, were performed in the NPT ensemble for a total of 15.2 μs of simulated time over all systems.

In order to test if systems with naturally occurring nucleobases are microscopically stable, we have analyzed the values of the first derivative of the natural logarithm of activity of the nucleobase, ln *a_N_*, with respect to natural logarithm of nucleobase molar fraction, ln *X_N_*, in the simulated systems (45–47). The quantity:

(1) \begin{equation*} \left( {\frac{{\partial {\rm ln}a_N }}{{\partial {\rm ln}X_N }}} \right) = \frac{1}{{1 + \rho _N X_N (G_{{NN}} + G_{{WW}} - 2G_{{ NW}} )}} \end{equation*}

where *G_NN_*, *G_NW_* and *G_WW_* denote Kirkwood–Buff integrals derived from nucleobase/nucleobase, nucleobase/water and water/water radial distribution functions (RDFs), respectively, must be positive for a system to be microscopically stable. Here, ρ_N_ stands for the nucleobase density number. The Kirkwood–Buff integrals were calculated using the following formula (45–47):

(2) \begin{equation*} G_{{SS}} (r) = 4\pi \int\limits_0^r {r'^2 [g_{{ss}} (r') - 1]dr'} \end{equation*}

where *g_SS_* denotes nucleobase/nucleobase (*g_NN_*), nucleobase/water (*g_NW_*) or water/water (*g_WW_*) RDFs, respectively, from which corresponding Kirkwood–Buff integrals (*G_NN_*, *G_NW_*, *G_WW_*) were derived. As anchor points for RDFs, we used centers of mass of nucleobases and water molecules. Finally, as representative examples for each individual nucleobase type, we have performed the above analysis on simulated systems with Leu residues and the results are reported in Figure 1B and Supplementary Table S2.

To quantify the interaction propensity of amino acids or sidechain analogs for different nucleobases, we have analyzed their behavior in the simulated mixtures both structurally and energetically. For simplicity, we describe the procedure for sidechain analogs only, but analogous calculations were also performed for all amino acid-containing systems. For structural analysis, RDFs were calculated by using as anchor points the centers of mass of amino acid sidechain analogs, nucleobases and water molecules. For energetic analysis, we have calculated differences between the total force-field potential energies corresponding to sidechain analog-nucleobase (*E_X-N_*) and sidechain analog-water interactions (*E*_*X*-*W*_):

(3) \begin{equation*} \Delta E_{{NW}_X } = E_{X \hbox{-} N} - E_{X \hbox{-} W} \quad {\rm [kJ/mol]}. \end{equation*}

Moreover, the obtained differences in potential energy between sidechain analog-nucleobase-water interactions ($\Delta E_{NW_X }$) were further subtracted between systems with different nucleobases (*N*_1_, *N*_2_) in order to obtain relative interaction propensities or preferences of each sidechain analog for a specific nucleobase with respect to other nucleobases ($\Delta E_{N_1 N_{2X} }$):

(4) \begin{equation*} \Delta E_{N_1 N_{2X} } = \Delta E_{N_1 W_X } - \Delta E_{N_2 W_X } \quad {\rm [kJ/mol]}. \end{equation*}

In a related study (M. Hajnic, J. I. Osorio and B. Zagrovic, unpublished data), we have simulated amino acids in the presence of only one type of nitrogenous base (unsubstituted pyrimidines or purines) in water solution as here, but also in mixed systems with both nitrogenous bases (unsubstituted pyrimidines and purines) present at the same time. The relative amino acids’ interaction propensities derived from mixed systems where both bases were present at the same time and those derived from differences between individual systems correlate with each other with a Pearson correlation coefficient *R* = 0.98. This suggests that one can obtain relative interaction propensities of amino acids for different nucleobases from individual interaction propensities derived from systems with only one nucleobase type present.

To be able to compare systems with slightly different molar compositions, the calculated potential energies between sidechain analog and water molecules were rescaled before obtaining the interaction propensity scale in order to have all systems correspond to exactly 0.86 molar fraction water. When rescaling, we implicitly assumed that the few additional water molecules behave on average in the same way as the rest of the water molecules in the system and contribute to the overall sidechain analog-water potential energy proportionally to their number. Analogous structural and energetic analysis was performed for systems containing amino acids with interaction energies evaluated over all amino acid atoms. Amino acid and sidechain analog interaction propensity scales are given in Supplementary Table S7 in units of kJ/mol. Note, however, that the exact energetic values given in our scales depend strongly on the particular features of simulated systems (such as molar fraction of water or nucleobases), and as such should primarily be considered and analyzed in a relative sense.

The obtained scales were used as described in Hlevnjak *et al.* (36) in order to assess the correlation between protein interaction propensities for different nucleobases and the nucleobase content of their cognate mRNAs over the complete *Homo sapiens, Escherichia coli* and *Methanocaldococcus jannaschii* proteomes. In the case of sidechain analog interaction propensity scales, glycines and prolines were ignored on the protein side together with their codons on the side of mRNA. The sequence datasets were extracted from the UniProtKB database (April 2013 release) as described previously (36,48). Window-averaged profiles of individual mRNAs and proteins were calculated in the same way as reported previously (36), where each position in the profile corresponds to the average value of the property in question over a window (with the size of 21 residues for proteins and 63 bases for mRNAs) centered at that position. As shown before (36), for window sizes anywhere between 10 and 40 residues, the results depend only marginally on window size (variation < 2%).

To test the significance of median values of profile-matching Pearson *R* distributions calculated for complete proteomes, we generated 10^6^ random scales and compared the medians of their profile-matching Pearson *R* distributions to the tested ones for each individual proteome. Random scales were generated by drawing numbers from a uniform distribution between 0 and 1. Finally, the *P*-values were calculated as the fraction of random scales whose medians of the profile-matching Pearson *R* distributions were greater than or equal to the tested ones in absolute value.

## RESULTS

### Validation and analysis of binding propensity scales

Natural nucleobases have low water solubility (49), ranging from 1.04 g/l for ADE to 8 g/l for CYT, corresponding to base molar fractions of $X_{{\rm ADE}} = 1 \times 10^{ - 4}$ and $X_{{\rm CYT}} = 1 \times 10^{ - 3}$, respectively. In order to: (i) realistically model nucleobase density at typical RNA–protein interfaces and (ii) reach a critical number of nucleobases that would allow us to observe interactions with amino acids or their sidechain analogs on a reasonable timescale, we have simulated systems whose nucleobase concentrations were significantly higher than their macroscopic solubility levels (e.g. $X_N = 0.14$). Practically speaking, we have simulated the behavior of amino acids and their sidechain analogs in hydrated, dynamic agglomerates of nucleobases as illustrated in Figure 1A for the Leu sidechain in CYT solution. While such systems, in fact, better approximate the effective concentration of nucleobases at typical RNA–protein interfaces, it was critical to first assess their thermodynamic stability at the microscopic level.

The stability of a binary, high-concentration mixture of water and nucleobases can be studied by analyzing the first derivative of the natural logarithm of activity of the nucleobase with respect to the natural logarithm of the nucleobase mole fraction, ∂ln *a_N_*/∂ln *X_N_* (45–47). This value, which should be positive for systems to be microscopically stable, was calculated from Equation (1) where *G_NN_*, *G_NW_* and *G_WW_* denote Kirkwood–Buff integrals derived from nucleobase/nucleobase, nucleobase/water and water/water RDFs, respectively (45–47). A typical set of such RDFs encountered in our simulations is given in Figure 1B (top panel) for the Leu sidechain in CYT solution. Importantly, due to the poor convergence of Kirkwood–Buff integrals, as an estimate of *G_SS_*, in all cases we took the average of *G_SS_* over distances starting from 1.5 nm (Figure 1B, lower panel). Following the above procedure, we could indeed show that the above requirement (i.e. ∂ln *a_N_*/∂ln *X_N_* > 0) is fulfilled for all four nucleobase types (Supplementary Table S2). This suggests that although our systems would over long timescales likely result in a creation of macroscopic aggregates, they are thermodynamically stable on the size- and time scales examined here and could be used as model systems to study the behavior of amino acids and their sidechain analogs in aqueous solutions of nucleobases. The fact that despite high nucleobase concentrations we did not observe formation of any static precipitates further corroborates this claim.

We have used our simulations to calculate differences between the total force-field potential energies corresponding to amino acid–nucleobase and amino acid–water interactions (and the same for sidechains). How do these energy-based interaction propensity scales compare with experimental results? The experimental PR scale (8), derived by analyzing the chromatographic mobility of amino acids in water mixtures of substituted pyridines such as dimethylpyridine (DMP), is one of the few examples where interactions between amino acids and nitrogenous bases have been systematically explored in experiment. Specifically, PR of a given amino acid was defined as the slope of a linear fit between the logarithm of its retention coefficient *R* and the logarithm of mole fraction of water in the pyridine–water solvent. In a related study (M. Hajnic, J. I. Osorio and B. Zagrovic, unpublished data), we have performed MD simulations of amino acids and their sidechain analogs in water/DMP mixtures using the same setup as here. The energy-based DMP/amino acid and DMP/sidechain analog interaction propensity scales derived from MD agree closely with the experimental PR scale (8) with Pearson *R* coefficients of 0.93 and 0.95, respectively, attesting to the general quality of our simulation methodology (M. Hajnic, J. I. Osorio and B. Zagrovic, unpublished data).

**Figure 2.**
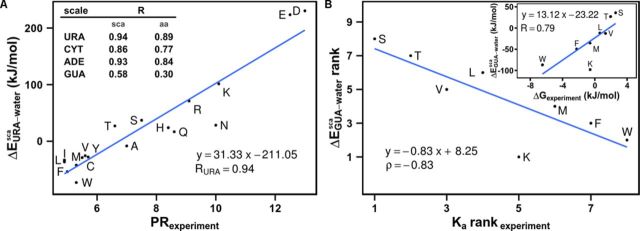
(**A**) Correlation between the experimentally derived polar requirement (PR_experiment_) scale (8) and the energy-based scale of sidechain analog interaction propensities for URA (in kJ/mol) obtained by simulation. Inset: Pearson correlation coefficients *R* between all sidechain analog (second column) and amino acid (third column) propensity scales and the PR scale. (**B**) Rank-order correlation between experimentally measured amino acid–guanosine association constants (10), and the computationally derived sidechain analog interaction energy scale for GUA (in kJ/mol). Inset: correlation between binding free energies (in kJ/mol) at the standard reference concentration of 1 M, as derived from association constants, and the the computationally obtained sidechain analog interaction energy scale for GUA (in kJ/mol).

Remarkably, the experimental PR scale (8) also exhibits close correlation with the energy-based amino acid interaction propensity scales derived here for URA (Pearson *R* = 0.89), ADE (*R* = 0.84) and CYT (*R* = 0.77), with a significantly weaker correlation observed for GUA (*R* = 0.30) (Figure 2A, inset table, third column). What is more, all of these correlations against the experimental PR scale improve even further if one uses sidechain analog scales instead (Figure 2A, inset table, second column), with the URA interaction propensity scale exhibiting the strongest correlation (*R* = 0.94), followed by ADE (*R* = 0.93), CYT (*R* = 0.86) and, finally, GUA (*R* = 0.58). In Figure 2A, we plot the sidechain analog scale for URA, a nucleobase which is physicochemically and sterically most similar to DMP, against the experimental PR scale (8) and the two exhibit remarkable similarity. Although the experimental PR scale and the computational URA, ADE and CYT scales were derived in very different ways, the close agreement between can be taken as evidence of the quality of the MD force field and the general computational methodology used. Moreover, such agreement also suggests that when it comes to capturing nucleobase/amino acid interaction specificity, DMP is actually a good model not only for naturally occurring pyrimidine bases URA and CYT, but also purine ADE.

When we compare our sidechain analog interaction propensities for GUA with the only analogous, extensive scale available from experiment, that of amino acid–guanosine binding constants for eight amino acids (Ser, Thr, Val, Leu, Met, Lys, Phe and Trp) (10), we obtain a Spearman rank-order correlation coefficient of ρ = −0.83 (Figure 2B) and a direct Pearson correlation coefficient of *R* = 0.79 when the association constants are converted to binding free energies (Figure 2B, inset). Interestingly, in our simulations we not only correctly capture the relative interaction propensities of aromatic sidechain analogs for GUA, but we also observe the same propensity trends as in the experiment for the relatively similar residues such as Ser and Thr or Val and Leu. What is more, if one excludes the outlier Lys, the rank correlation increases to ρ = −0.96. On the other hand, the level of correlation drops significantly if one uses the computational scale for amino acids, here also including the value for Gly (ρ = −0.62 and *R* = 0.46). Finally, the experimentally derived binding free energies of four amino acids for adenosine (Val, Lys, Phe, Trp) (ρ = −0.80 and *R* = 0.52) and two for cytidine (Phe, Trp) (10) show the same trend as observed in our sidechain analog interaction propensity scales for the equivalent bases, with similar results for amino acid scales (ρ = −0.80 and *R* = 0.50 for the adenosine case). Overall, a combination of the above thermodynamic stability analysis and the favorable comparison with experiment reassuringly suggests that the essential physical chemistry behind amino acid/nucleobase interactions remains approximately the same even at relatively high nucleobase concentrations as studied here. This furthermore suggests that our simulation-based scales can be used to greatly extend the limited experimental data available and characterize interactions with nucleobases for all amino acids and sidechain analogs. Interestingly, in many cases, our simulations with sidechain analogs match the experimental data obtained with amino acids slightly better than the simulations with amino acids themselves (Figure 2), a finding we do not currently have a full explanation for. A part of the reason may be that the GROMOS53A6 force field was parameterized to match solvation free energies of sidechain analogs in cyclohexane or water and not those of complete amino acids. It is possible that a potentially lower accuracy of parameters for complete amino acids may be responsible for a greater discrepancy from experiment in that case. However, as sidechain analogs capture the behavior of protein residues at RNA–protein interfaces arguably better than the zwitterionic amino acids do, in the remainder of this text we primarily focus on sidechain analogs, while always giving the results for amino acids as a point of comparison.

**Figure 3.**
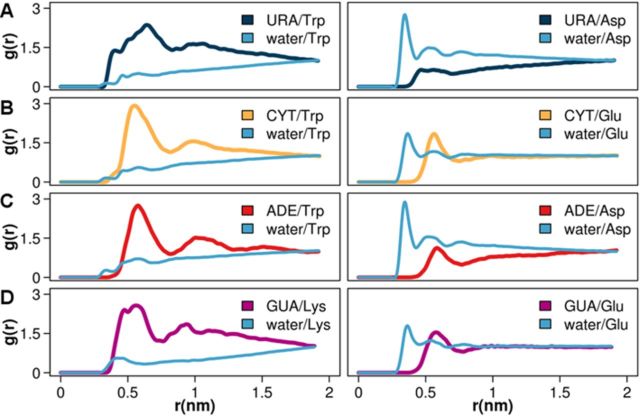
Water/sidechain-analog and nucleobase/sidechain-analog radial distribution functions *g*(*r*) for the most favorable (left column) and the least favorable (right column) sidechain analog interacting partners for the four RNA nucleobases, as determined by energy-based interaction propensity scales for: (**A**) URA, (**B**) CYT, (**C**) ADE and (**D**) GUA.

The above energetic analysis is well illustrated by a structural exploration using RDFs. In Figure 3, we show water/sidechain-analog and nucleobase/sidechain-analog RDFs for the most favorable and the least favorable interacting partners of the four RNA nucleobases, as determined by the analysis of interaction energies. URA, CYT and ADE, for example, all exhibit the strongest preference for interacting with Trp relative to all other residues, which is illustrated by the presence of a pronounced first peak in their nucleobase/Trp RDFs. On the other hand, in the case of GUA the strongest favorable interactions are seen for Lys. When it comes to the least favorable interactions, in all cases they are invariably seen with the negatively charged Glu and Asp. The presence of a well-defined peak in CYT/Glu, ADE/Asp and GUA/Glu RDFs, however, suggests that, although unfavorable, some of these interactions do exhibit a sizable level of structural organization. Nonetheless, for all energetically unfavorable interactions, it is clear that the residues in question prefer to interact with and be surrounded by water molecules, as indicated by strong, well-defined first peaks in the respective water/sidechain-analog RDFs.

**Figure 4.**
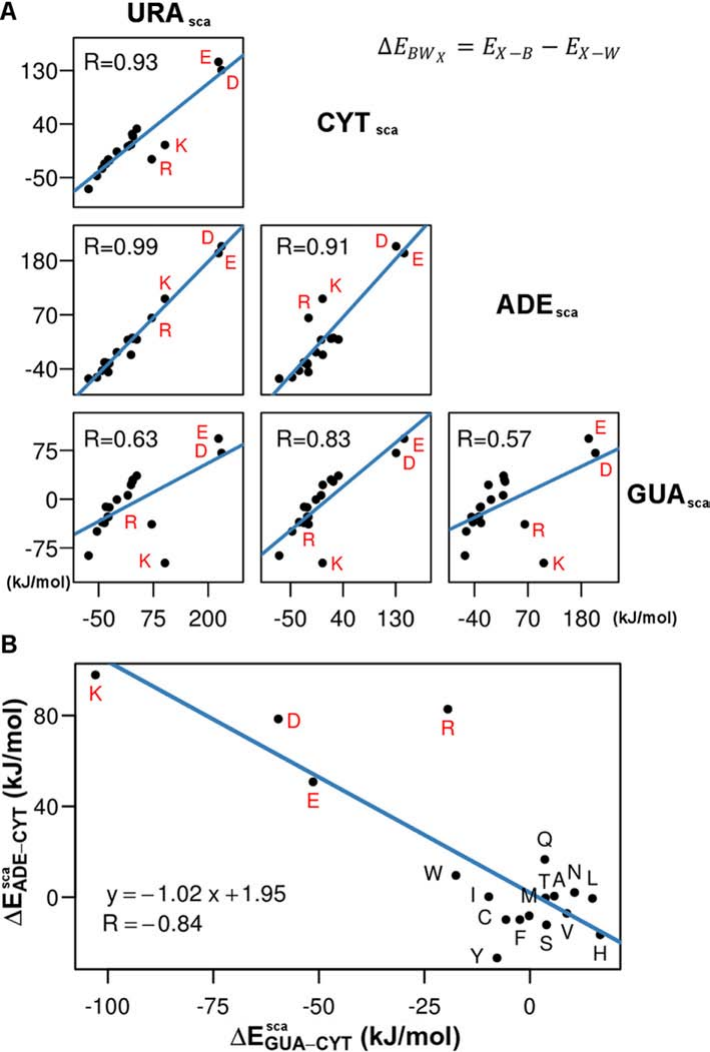
(**A**) A direct comparison between energy-based sidechain analog interaction propensity scales for the four nucleobases with Pearson correlation coefficients given in the graphs. In each graph, the four charged amino acids are labeled in red. (**B**) Correlation between relative energy-based sidechain analog GUA–CYT and ADE–CYT interaction propensity scales (in kJ/mol) derived from simulations of different systems.

As discussed above, the GUA-based interaction energy scales differ most from all other scales. When correlating the individual scales against each other, we indeed find that the GUA scale deviates most from other scales, which is primarily due to the behavior of charged sidechain analogs (Figure 4A). Namely, in the GUA/water mixture, Lys and Arg exhibit lower interaction energies with GUA than with water molecules, which is not the case in any other nucleobase/water systems except for the CYT/Arg system (Figure 4A). Furthermore, Asp and Glu also exhibit significantly more favorable interaction energies with GUA as compared to other energy-based interaction propensity scales (Figure 4A). Although in absolute terms these two anionic sidechains do not interact favorably with GUA (i.e. they exhibit positive energies), the extent of this unfavorable bias is the least as compared to other bases (Figure 4A). Similar results are also seen in the simulations with complete amino acids (data not shown).

A particularly telling comparison in this regard concerns the behavior of GUA- and ADE-based scales. If one, for example, examines relative energy-based interaction propensity scales, one observes a remarkable asymmetry in the behavior of GUA and ADE (Supplementary Figure S1). In particular, the relative ADE–CYT scale is strongly inversely correlated with those involving GUA (GUA–CYT, *R* = −0.84 and GUA–URA, *R* = −0.95) with no significant correlations or anti-correlations for the ADE–URA relative scale (Supplementary Figure S1). In Figure 4B, we illustrate this difference in the case of GUA–CYT and ADE–CYT relative scales and it is clear that the effect is completely due to the nature of the interactions of the charged residues with ADE and GUA relative to that with CYT. While GUA strongly prefers to interact with Lys, Arg, Asp and Glu as compared to CYT (with, for example, $\Delta E_{{\rm GUA} \hbox{--} {\rm CYT}}^{{\rm sca}}$ of cca. −100 kJ/mol in the case of Lys), ADE almost equally strongly prefers not to interact with these residues (with $\Delta E_{{\rm ADE} \hbox{--} {\rm CYT}}^{{\rm sca}}$ of cca. 100 kJ/mol in the case of Lys) again as compared to CYT (Figure 4B). This effect clearly demonstrates the paramount importance of specific ring substituents especially in the case of purine bases, which was already observed in our analysis of knowledge-based nucleobase-residue interaction propensity scales (23). Interestingly, while the sidechain analog scale derived presently for ADE correlates reasonably well with the equivalent knowledge-based scale (Spearman ρ = 0.57 for the 2+ scale from Polyansky *et al.* (23)), the correlations for all other scales including the GUA scale are significantly weaker (|ρ| < 0.2) (Supplementary Table S3).A similar trend is also seen with amino acid interaction propensity scales (Supplementary Table S3). On the other hand, the relative scales of GUA derived presently for sidechain analogs agree somewhat better with those derived in the knowledge-based analysis (23) with, for example, GUA–URA and GUA–CYT correlating with Spearman ρ of 0.40 or 0.38, respectively (Supplementary Table S4). While these correlations between complete scales are relatively weak, it is important to mention that they agree much better when it comes to the relative placement of charged residues only, which is in the end chiefly responsible for the qualitative similarities between the scales, as discussed below.

### Analysis of the mRNA-cognate protein complementarity hypothesis

**Figure 5.**
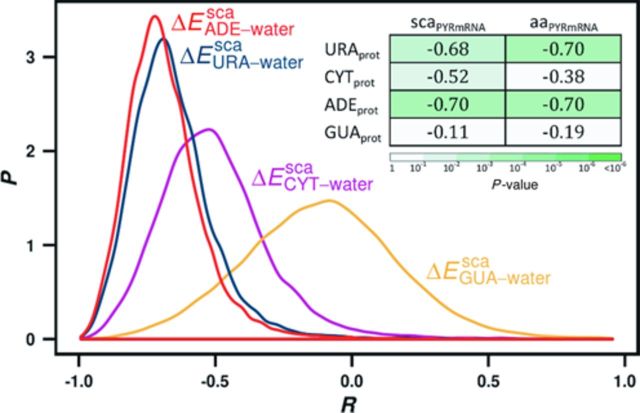
Distributions of Pearson correlation coefficients between window-averaged PYR content profiles of mRNAs and their cognate proteins’ profiles of interaction propensity for different RNA nucleobases (URA, CYT, ADE and GUA) assessed using computationally derived sidechain analog scales. Inset: median values of distributions of Pearson correlation coefficients between window-averaged PYR content profiles of mRNAs and their cognate proteins’ profiles of interaction propensity for different RNA nucleobases, calculated over the entire human proteome (sidechain analogs, ‘sca,’ and complete amino acids, ‘aa’).

We have used the obtained scales to study the relationship between the nucleobase content of mRNA coding sequences and the nucleobase interaction propensities of their cognate protein sequences for the entire *H. sapiens, M. jannaschii and E. coli* proteomes. We have performed this analysis by comparing window-averaged sequence profiles of the two cognate biopolymers as elaborated before (23,36,37), whereby one obtains a Pearson *R* for each cognate pair, i.e. a distribution of Pearson *R*s over the whole proteome. Note that negative correlations here denote a positive relationship between nucleobase content and interaction propensity, which comes from the fact that propensity is defined using an energy scale (the lower the energy, the higher the propensity). Our results show that PYR density profiles of mRNAs quantitatively match the energy-based URA-, CYT- and ADE-interaction propensity profiles of their cognate protein sequences across the entire human proteome, with no significant correlation being observed for GUA scales, as demonstrated for *H. sapiens* in Figure 5. More specifically, the median correlation coefficients for URA, CYT and ADE sidechain-based scales are −0.68, −0.52 and −0.70, respectively, while for the GUA scale this value drops to −0.11 (Figure 5, inset). For *M. jannaschii*, the median correlation coefficients of mRNA–protein pairs are as high as those observed for the human proteome or higher, while for *E. coli* the values are slightly lower, but still statistically significant (Supplementary Figures S2A and S3A). Similar values are also seen for amino acid-based scales as well. In other words, PYR-rich regions in mRNAs tend to code for regions in their cognate proteins that exhibit more favorable interaction energies with URA, CYT and ADE relative to water as compared to the PUR-rich regions. Interestingly, the correlation coefficients obtained for mRNA density profiles of individual bases are significantly weaker (Supplementary Table S5), as was already observed before (23,36).

**Figure 6.**
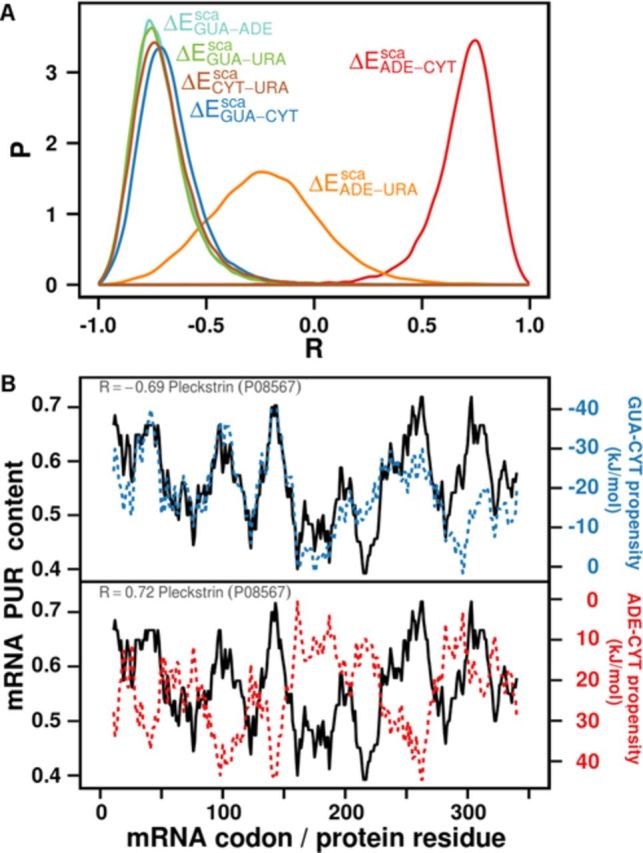
(**A**) Distributions of Pearson correlation coefficients between window-averaged PUR content profiles of mRNAs and their cognate proteins’ profiles of relative interaction propensity for different combinations of RNA nucleobases, calculated over the entire human proteome. The propensities were obtained from the energetic analysis of different systems from MD simulations. (**B**) Typical profiles of mRNA PUR content and protein sequence interaction propensity calculated using the computationally derived sidechain analog GUA–CYT and ADE–CYT relative interaction propensity scales. The two examples were chosen because their Pearson R coefficients correspond to the medians over the respective distributions over the complete human proteome.

As mentioned above, the GUA scale does not yield any significant correlation with PYR, i.e. PUR content on the side of mRNA. However, analysis of relative scales reveals that mRNA PUR density profiles closely and quantitatively match their cognate protein profiles capturing the relative preference of residues to interact with GUA relative to all other nucleobases. For example, protein sequence profiles of relative GUA–CYT binding propensities match their cognate mRNA PUR density profiles with the median Pearson *R* = −0.69 (*P*-value = 1 × 10^−3^) over the entire human proteome (Table 1). What this means is that one half of all mRNA-cognate protein pairs in the human proteome display profile matching that is equal or better than the median representative, Pleckstrin (P08567) shown in Figure 6B. Interestingly, though, much weaker correlation is seen if instead of PUR content, which is up to a constant equivalent to PUR–PYR content, one here analyzes GUA–CYT content along mRNA (median *R* = −0.48). Similar results are also obtained for the relative GUA–URA and GUA–ADE scales (*P*-values 3 × 10^−4^ and 4 × 10^−4^, respectively) (Table 1). On the other hand, the ADE–CYT scale results in a similar level of matching, but now when it comes to mRNA PYR-density mRNA. In Figure 6B, we illustrate this in the case of the median representative protein Pleckstrin (P08567) and its mRNA. Interestingly, the ADE–URA scale exhibits no significant correlation whatsoever, while the CYT–URA scale exhibits a significant level of matching with mRNA PUR density profiles (*P*-value = 4 × 10^−4^) (Table 1). Again, we observe the same trend as with non-relative interaction propensity scales (Supplementary Table S5) that the correlation coefficients for mRNA density profiles of individual base are weaker than the mRNA PUR density profiles (Supplementary Table S6). The same trends seen for human proteome extend to other organisms as well (Supplementary Figures S2B and C and S3B and C). As the main reason for the matching detected in our analysis is the genetic code, which is the same for all the organisms studied, it is not surprising that one obtains similar levels of matching no matter in which organism one looks. The differences, on the other hand, can be attributed to the exact mRNA and protein composition in individual proteomes. Finally, qualitatively identical results are obtained for systems with zwitterionic amino acids instead of sidechain analogs (Table 1).

**Table 1.**
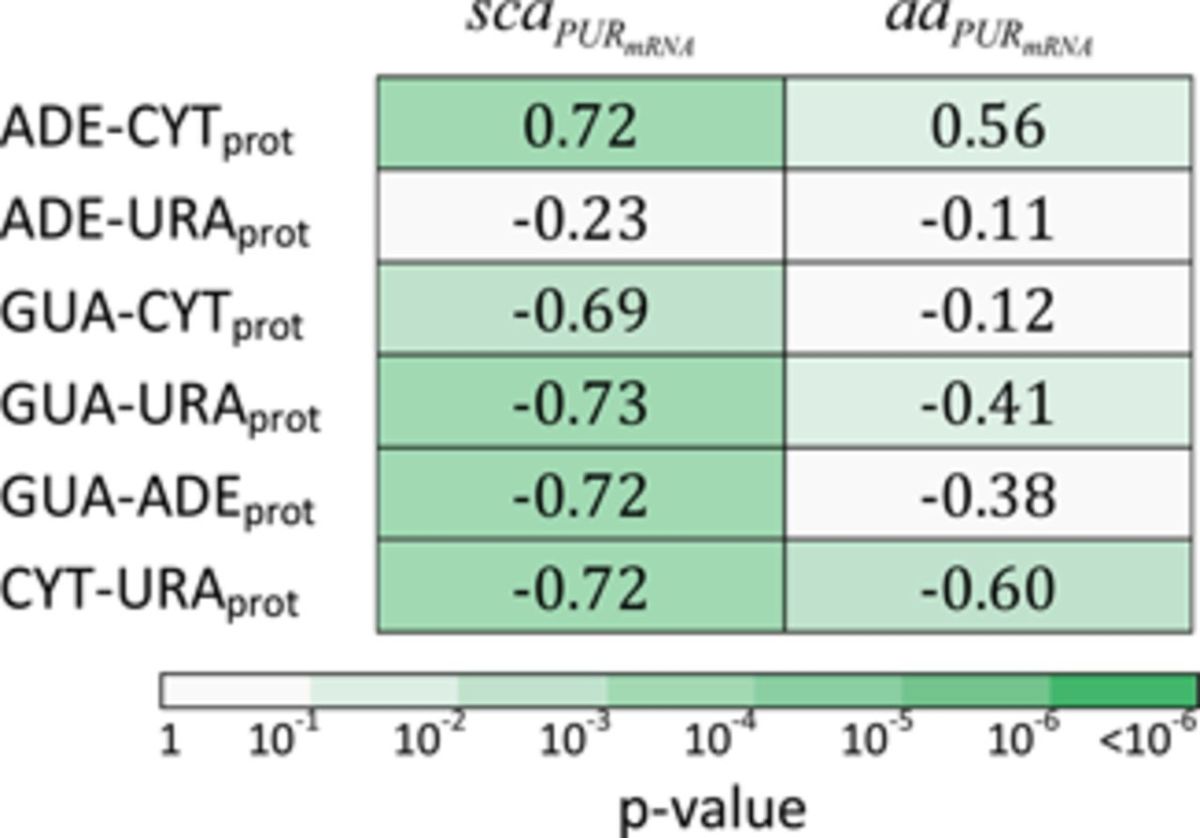
Median values of distributions of Pearson correlation coefficients between window-averaged PUR content profiles of mRNA molecules and their cognate proteins’ profiles of relative interaction propensities for nucleobases calculated over the entire human proteome. The interaction propensities were obtained from the energetic analysis of both sidechain (sca) and amino acid (aa) containing systems.

## DISCUSSION

In the present study, we have for the first time systematically analyzed the behavior of amino acids and their sidechain analogs in high-concentration aqueous solutions of naturally occurring RNA nucleobases. Our results show that amino acids and their sidechain analogs display highly differentiated interaction propensities for different nucleobases depending on the ring architecture and, even more importantly, ring substituents. It is our hope that these scales will provide: (i) a rigorous, quantitative, physicochemical foundation for rationalizing the specificity in RNA–protein interactions in different contexts, and (ii) a powerful tool for sculpting and modifying such specificity for biomedical and bioengineering purposes.

As discussed above, our simulations were carried out at nucleobase concentration levels exceeding the experimentally known solubility limits. However, a strong agreement with extant experimental data, a general absence of stable aggregates and favorable results of thermodynamic stability analysis all suggest that the simulated model systems do capture the essential features of amino acid/nucleobase interactions even at high concentrations. Moreover, even if the simulated systems would over time move in the direction of precipitation, the partitioning of amino acids and their sidechain analogs between water- and base-rich fractions occurs much more quickly, allowing one to accurately capture interaction propensities with relatively short simulations. Finally, the number of water molecules in our simulations was such that for each base there was enough water to account for one full hydration shell around it. As such, our simulated systems in all likelihood better approximate the situation at typical hydrated RNA–protein binding interfaces than would more dilute solutions.

Our analysis of energy-based interaction preferences was based on a critical assumption that the potential energies between amino acids or sidechain analogs and nucleobases or water accurately capture the free energies of these interactions. In other words, we assumed that it is primarily the enthalpic part of free energy that is responsible for the relative difference in amino acid–nucleobase interactions, with the entropic component being proportional to it. A similar assumption was made by Stumpe and Grubmüller in their study of amino acid interactions with urea and their influence on protein folding (50). In a related study (A. de Ruiter and B. Zagrovic, in preparation), we have used MD simulations and umbrella sampling to evaluate absolute binding free energies between nucleobases and amino acid sidechain analogs in water. By comparing the sidechain analog interaction propensities derived in this work to the absolute free energies, which fully account for both enthalpy and entropy, we observe a high level of correlation with Spearman correlation coefficients of ρ = 0.88 (URA), ρ = 0.85 (CYT), ρ = 0.62 (GUA) and ρ = 0.88 (ADE) (A. de Ruiter and B. Zagrovic, in preparation). Although the behavior of amino acids or their sidechain analogs in crowded solutions of nucleobases need not necessarily match that with only one nucleobase present, such a high level of correlation does support the existence of a strong relationship between free energies and their enthalpic components in the former case. Finally, the fact that the obtained scales agree well both with experimental results (8,10) as well as with the structural analysis of intermolecular contacts (23) lends further support to this claim.

Here, we have used the derived interaction propensity scales to investigate how the relationships observed at the level of amino acids and nucleobases translate to the level of complete coding sequences of mRNAs and their cognate proteins. Our central aim was to further examine the recently proposed complementarity hypothesis and its relationship with the structure and the origin of the genetic code (23,36,37). In accordance with our results obtained using knowledge-based potentials (23), we have observed that the higher the pyrimidine content of mRNAs, the higher the propensity of their cognate proteins' propensities to interact with URA, CYT and ADE, but importantly not with GUA (Figure 5). Actually, the fact that GUA- and ADE-based scales exhibit opposite behavior when it comes to their relationship with PYR-based scales (Supplementary Figure S1) suggests that the key element in determining the specificity of interaction between amino acids and nucleobases is not the nature of the heterocyclic ring, but rather that of ring substituents. In particular, our present results suggest that this difference stems primarily from the behavior of charged amino acids, which is reasonable considering the fact that the two purine bases are largely isosteric and differ primarily when it comes to ring substituents and their charge distribution. This is also supported by a related analysis in which we showed that unsubstituted purine and pyrimidine rings result in highly correlated scales when it comes to their interactions with amino acids (M. Hajnic, J. I. Osorio and B. Zagrovic, unpublished data).

In support of this reasoning, we have observed a strong relationship between the average PUR content of mRNA sequences and the relative preference of their cognate protein sequences to interact with GUA relative to other bases (Figure 6A, Table 1). In accordance with the stereochemical hypothesis and our generalizations of it (23,36,37), GUA exhibits strong preference for interaction with PUR-coded amino acids relative to all other bases. Importantly, this effect appears to be primarily due to the behavior of charged amino acids Glu, Asp, Arg and Lys. These results are fully consistent with our previous knowledge-based analysis of residue preferences for different nucleobases: there, GUA interaction preferences on the side of amino acids or proteins correlated extremely well with purine density at the side of their cognate codons or mRNA, while ADE interaction preferences were much closer to those of pyrimidine bases (23), as also seen here. While the full biological meaning of this result still remains to be elucidated, we are confident that it represents an important principle concerning the mRNA–protein relationship in general. Overall, our results give support to the generalized stereochemical hypothesis of the origin of the genetic code, in which GUA plays the role of an archetypal purine (i.e. purine richness on the side of codons or mRNAs parallels high levels of relative guanine interaction propensity on the side of cognate amino acids or proteins), while the opposite is seen for CYT, URA and ADE (i.e. pyrimidine richness of mRNA mirrors high relative interaction propensity for these nucleobases when it comes to cognate amino acids or proteins) (23,36,37). In this context, the presence of adenines negatively affects complementarity levels, as discussed before (37). Intriguingly, despite the fact that the propensity scales were derived for specific bases, the highest levels of matching are observed if one considers PUR (i.e. PYR) density on the side of mRNA and not that of individual bases (Supplementary Tables S5 and S6). This effect, which was already observed before (23,36,37), still requires a full explanation. However, we believe it suggests that the core of the genetic code was originally defined at the level of a coarse-grained nucleobase alphabet in which differences between specific purines, i.e. pyrimidines, were not critical.

Our results with specifically Glu and Asp and their interactions with GUA show that the most basic version of the stereochemical hypothesis, the one in which the genetic code evolved on the basis of direct interactions between amino acids and their codons, can at best hold for a subset of amino acids only. In particular, Glu and Asp do not appear to favorably interact with any nucleobases in the aqueous environment, although in our knowledge-based analysis (23) they do show a strong preference for interacting with purine bases and especially GUA. The solution to this seeming paradox is provided by our present results: although the negatively charged Glu and Asp do not show direct preference for binding to GUA, they appear to be the least unfavorable interacting partners for GUA when compared to all other nucleobases. It is very possible that the preferences one sees in the knowledge-based analysis of known protein–RNA complexes are in part a consequence of such a negative selection. One way in which this result could be made consistent with the stereochemical hypothesis and especially its generalized version, even for Glu and Asp, is if one assumes that the genetic code evolved in a context in which the role of the translation apparatus was not to link individual amino acids according to the mRNA template, but rather short peptides. In this scenario, other amino acids would provide the source of favorable binding free energy to the mRNA template, while Glu and Asp would contribute to the specificity of binding only.

Overall, our study shows that by using MD simulations and extensive sampling we can distinguish between amino acid or sidechain analog interaction propensities for different nucleobases. Remarkably, the interaction propensities derived from simulations of individual monomers yield close correspondences at the level of complete proteins and mRNA molecules, giving support to the mRNA/protein complementarity hypothesis as recently proposed. Although our present results are highly suggestive, it should be nonetheless emphasized that the only rigorous test of the complementarity hypothesis can come from direct experimental work. We hope that our present results will serve not only as a source of motivation in this direction, but also as a foundation for different computational and experimental studies of RNA/protein interactions in general (51–53).

## SUPPLEMENTARY DATA

Supplementary Data are available at NAR Online.

SUPPLEMENTARY DATA
