# Supplementary material for: Computational analysis of amino acids and their sidechain analogs in crowded solutions of RNA nucleobases with implications for the mRNA–protein complementarity hypothesis
Source: Nucleic Acids Res. 2014 Oct 31;42(21):12984–94. doi: 10.1093/nar/gku1035 (PMC4245939; doi:10.1093/nar/gku1035)
Supplement: SUPPLEMENTARY DATA [file supp_42_21_12984__index.html]

Computational analysis of amino acids and their sidechain analogs in crowded solutions of RNA nucleobases with implications for the mRNA–protein complementarity hypothesis — Computational analysis of amino acids and their sidechain analogs in crowded solutions of RNA nucleobases with implications for the mRNA–protein complementarity hypothesis — SUPPLEMENTARY DATA 

# Computational analysis of amino acids and their sidechain analogs in crowded solutions of RNA nucleobases with implications for the mRNA–protein complementarity hypothesis

## SUPPLEMENTARY DATA

**Files in this Data Supplement:**

- SUPPLEMENTARY DATA
- SUPPLEMENTARY DATA
