# Supplementary material for: Computational analysis of amino acids and their sidechain analogs in crowded solutions of RNA nucleobases with implications for the mRNA–protein complementarity hypothesis
Source: Nucleic Acids Res. 2014 Oct 31;42(21):12984–94. doi: 10.1093/nar/gku1035 (PMC4245939; doi:10.1093/nar/gku1035)
Supplement: SUPPLEMENTARY DATA [file supp_gku1035_nar-01911-r-2014-File008.pdf]

submitted to Nucleic Acids Research

Supplementary Information

Computational analysis of amino acids and their sidechain analogs in crowded solutions of RNA nucleobases with implications for the mRNA-protein complementarity hypothesis

Matea Hajnic, Juan Osorio Iregui[#] and Bojan Zagrovic[*]

Department of Structural and Computational Biology, Max F. Perutz Laboratories, University of Vienna, Vienna 1030, Austria

September 2014

[#]present address: Juan Osorio Iregui, Institute for Theoretical Physics, ETH Zürich 8093, Switzerland

[*]to whom correspondence should be addressed. Tel: +43 1 4277 9522; Fax: +43 1 4277 9522; Email: bojan.zagrovic@univie.ac.at

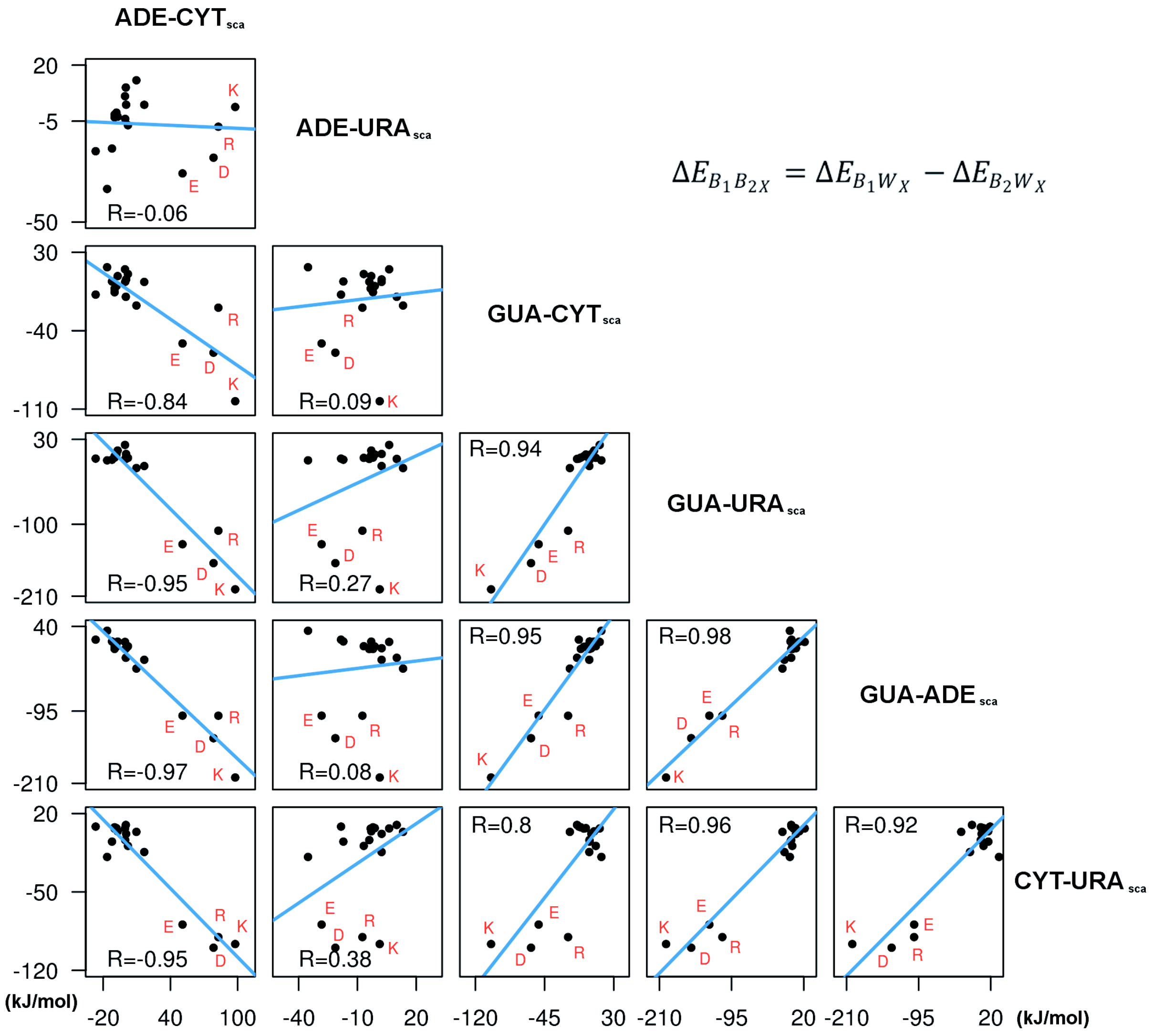

$$\Delta E_{B_1 B_{2X}} = \Delta E_{B_1 W_X} - \Delta E_{B_2 W_X}$$

Figure S1. Graphical representation of correlations between different relative energy-based sidechain analog interaction propensity scales with the associated Pearson correlation coefficients. In each graph, the four charged amino acids are labeled in red.

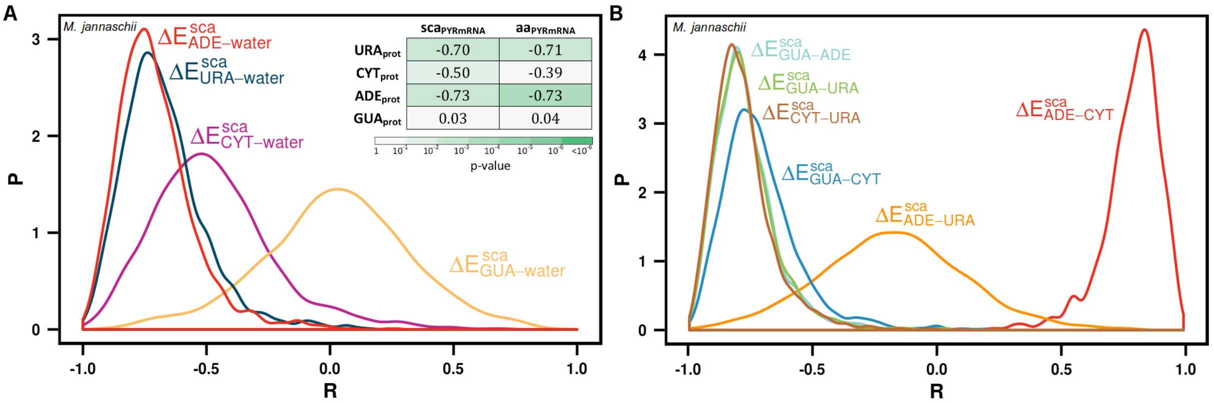

C

	ADE-CYT$_{prot}$	ADE-URA$_{prot}$	GUA-CYT$_{prot}$	GUA-URA$_{prot}$	GUA-ADE$_{prot}$	CYT-URA$_{prot}$
**sca$_{PURmRNA}$**	0.81	-0.18	-0.74	-0.79	-0.79	-0.80
**aa$_{PURmRNA}$**	0.63	-0.03	-0.32	-0.55	-0.53	-0.66

1 10^{-1} 10^{-2} 10^{-3} 10^{-4} 10^{-5} 10^{-6} $<10^{-6}$

p-value

Figure S2. (**A**) Distributions of Pearson correlation coefficients for *M. jannaschii* proteome between window-averaged PYR content of mRNAs and their cognate proteins' interaction propensities for different nucleobases (URA, CYT, ADE and GUA) assessed using computationally derived sidechain analog scales. Inset: median Pearson correlation coefficients of the distributions in the main panel together with their counterparts obtained for amino-acid scales (sidechain analogs, *sca*, and complete amino acids, *aa*). (**B**) Distributions of Pearson correlation coefficients for window-averaged profiles between PUR content of mRNA molecules and their cognate proteins' relative interaction propensities for different combinations of nucleobases as calculated for the whole *M. jannaschii* proteome. The propensities were obtained from the energetic analysis of different systems from MD simulations. (**C**) Median values of distributions of Pearson correlation coefficients for window-averaged profiles between PUR content of mRNA molecules and their cognate proteins' relative interaction-propensities for nucleobases shown in (**B**) for the whole *M. jannaschii* proteome. The interaction propensities were obtained from the energetic analysis of both sidechain (sca) and amino acid (aa) containing systems.

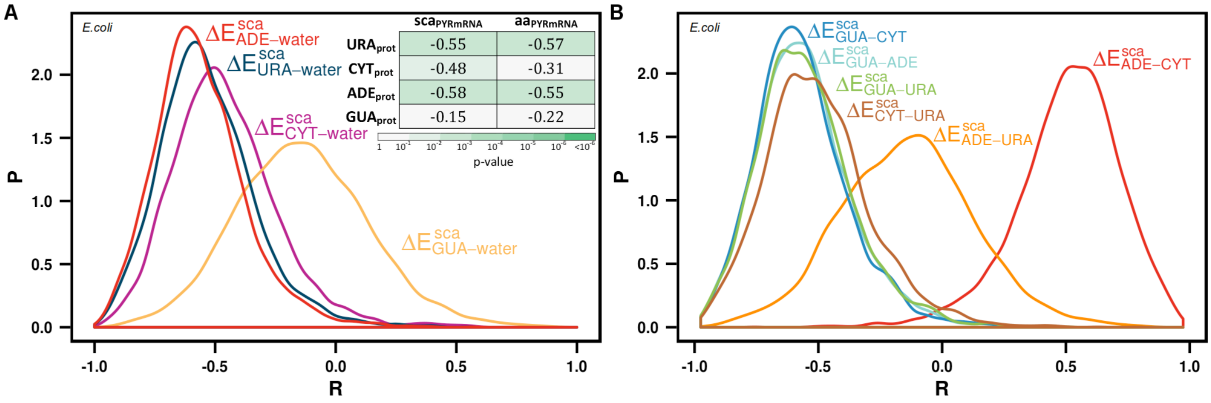

C

	$ADE\text{-}CYT_{prot}$	$ADE\text{-}URA_{prot}$	$GUA\text{-}CYT_{prot}$	$GUA\text{-}URA_{prot}$	$GUA\text{-}ADE_{prot}$	$CYT\text{-}URA_{prot}$
$sca_{PURmRNA}$	0.53	-0.14	-0.58	-0.57	-0.57	-0.52
$aa_{PURmRNA}$	0.42	-0.13	-0.04	-0.27	-0.24	-0.45

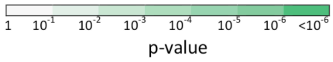

Figure S3. (**A**) Distributions of Pearson correlation coefficients for *E. coli* proteome between window-averaged PYR content of mRNAs and their cognate proteins' interaction propensities for different nucleobases (URA, CYT, ADE and GUA) assessed using computationally derived sidechain analog scales. Inset: median Pearson correlation coefficients of the distributions in the main panel together with their counterparts obtained for amino-acid scales (sidechain analogs, *sca*, and complete amino acids, *aa*). (**B**) Distributions of Pearson correlation coefficients for window-averaged profiles between PUR content of mRNA molecules and their cognate proteins' relative interaction propensities for different combinations of nucleobases as calculated for the whole *E. coli* proteome. The propensities were obtained from the energetic analysis of different systems from MD simulations. (**C**) Median values of distributions of Pearson correlation coefficients for window-averaged profiles between PUR content of mRNA molecules and their cognate proteins' relative interaction-propensities for nucleobases shown in (**B**) for the whole *E. coli* proteome. The interaction propensities were obtained from the energetic analysis of both sidechain (sca) and amino acid (aa) containing systems.

Table S1. Composition of MD boxes for simulated systems. In all simulation boxes, there was 1 amino acid or amino-acid sidechain analog (N_{AA}). The number of nucleobases (N_N) and water molecules (N_W) differs depending on the system. In the case of systems where all amino acids did not have the same number of water molecules, we report a range. The box content was the same for systems with amino-acid sidechain analogs.

System	N_{AA}	N_N	N_W
URA	1	180	1117-1120
CYT	1	180	1120
ADE	1	170	1026-1035
GUA	1	165	979-983

Table S2. Stability factors of systems with naturally occurring nucleobases as assessed from Kirkwood-Buff integrals: G_{NN}, G_{WW} and G_{NW}. The positive value of $\partial \mathit{ln}\ a_N/\partial \mathit{ln}\ X_N$ indicates that simulated systems are thermodynamically stable.

System	X_W	$V_{cell}(nm^3)$	$\rho_N(nm^{-3})$	$G_{NN}(nm^3)$	$G_{WW}(nm^3)$	$G_{NW}(nm^3)$	$\partial \mathit{ln}\ a_N/\partial \mathit{ln}\ X_N$
URA	0.86	57.76	3.12	6.86	3.31	-4.91	0.02
CYT	0.86	58.91	3.06	2.41	1.36	-1.96	0.05
ADE	0.86	57.98	2.93	4.49	3.34	-4.04	0.02
GUA	0.86	55.85	2.95	2.48	2.01	-2.41	0.04

W -> water; N -> nucleobase

Table S3. Spearman correlation coefficients between knowledge-based potential (KBP) scales from Polyansky et al. (2+ scales) (1) and energy-based sidechain analog or amino-acid (in parenthesis) interaction propensity scales obtained by simulations in this study for the four nucleobases.

	URA_{KBP}	CYT_{KBP}	ADE_{KBP}	GUA_{KBP}
**URA**	0.18 (0.16)	0 (0.04)	0.6 (0.57)	-0.43 (-0.45)
**CYT**	0.17 (0.33)	-0.12 (-0.15)	0.52 (0.22)	-0.29 (-0.18)
**ADE**	0.27 (0.21)	-0.05 (0.17)	0.57 (0.36)	-0.43 (-0.38)
**GUA**	0.02 (0.04)	-0.11 (0.22)	0.28 (0.17)	-0.11 (-0.23)

Table S4. Spearman correlation coefficients between relative knowledge-based potential (KBP) scales from Polyansky et al. (2+ scales) (1) and relative energy-based sidechain analog or amino-acid (in parenthesis) interaction propensity scales obtained by simulations in this study.

	$(ADE\text{-}CYT)_{KBP}$	$(ADE\text{-}URA)_{KBP}$	$(GUA\text{-}CYT)_{KBP}$	$(GUA\text{-}URA)_{KBP}$	$(GUA\text{-}ADE)_{KBP}$	$(CYT\text{-}URA)_{KBP}$
**ADE-CYT**	-0.1 (-0.26)	0.01 (0.08)	-0.55 (-0.45)	-0.47 (-0.17)	-0.49 (-0.19)	-0.04 (0.27)
**ADE-URA**	-0.18 (0.03)	-0.23 (-0.39)	-0.08 (0.26)	0.02 (0.02)	0.29 (0.25)	0.03 (-0.24)
**GUA-CYT**	0.26 (-0.14)	0.22 (0.04)	0.38 (-0.15)	0.29 (0.07)	0.12 (0.10)	-0.02 (0.28)
**GUA-URA**	-0.17 (-0.09)	-0.05 (-0.10)	0.31 (-0.02)	0.40 (0.05)	0.44 (0.20)	0.14 (0.16)
**GUA-ADE**	0.12 (-0.05)	0.21 (0)	0.45 (-0.04)	0.44 (0.06)	0.27 (0.15)	0.09 (0.20)
**CYT-URA**	-0.31 (0.16)	-0.14 (-0.21)	0.24 (0.43)	0.41 (0.14)	0.59 (0.23)	0.27 (-0.26)

Table S5. Median values of distributions of Pearson correlation coefficients for window-averaged profiles between nucleobase content of mRNA molecules and their cognate proteins' interaction propensities for nucleobases derived from **A)** sidechain analog (sca) and **B)** amino acid (aa) simulations as evaluated over the human proteome. The interaction propensities were obtained from the energetic analysis of simulated systems.

A)

	U_{mRNA}	C_{mRNA}	A_{mRNA}	G_{mRNA}	PYR_{mRNA}
URA^{sca}_{prot}	-0.49	-0.39	0.50	0.34	-0.68
CYT^{sca}_{prot}	-0.34	-0.29	0.39	0.26	-0.52
ADE^{sca}_{prot}	-0.51	-0.40	0.51	0.35	-0.70
GUA^{sca}_{prot}	-0.20	0.06	0.01	0.15	-0.11

1 10^{-1} 10^{-2} 10^{-3} 10^{-4} 10^{-5} 10^{-6} $<10^{-6}$

p-value

B)

	U_{mRNA}	C_{mRNA}	A_{mRNA}	G_{mRNA}	PYR_{mRNA}
URA^{aa}_{prot}	-0.42	-0.46	0.49	0.36	-0.70
CYT^{aa}_{prot}	-0.31	-0.18	0.29	0.17	-0.38
ADE^{aa}_{prot}	-0.37	-0.51	0.51	0.34	-0.70
GUA^{aa}_{prot}	-0.04	-0.18	-0.30	0.55	-0.19

1 10^{-1} 10^{-2} 10^{-3} 10^{-4} 10^{-5} 10^{-6} $<10^{-6}$

p-value

Table S6. Median values of distributions of Pearson correlation coefficients for window-averaged profiles between nucleobase content of mRNA molecules and their cognate proteins' relative interaction propensities for nucleobases derived from **A)** sidechain analog (sca) and **B)** amino acid (aa) simulations as evaluated over the human proteome. The interaction propensities were obtained from the energetic analysis of simulated systems.

A)

	U_{mRNA}	C_{mRNA}	A_{mRNA}	G_{mRNA}	PUR_{mRNA}
$ADE\text{-}CYT^{sca}_{prot}$	-0.51	-0.42	0.52	0.37	0.72
$ADE\text{-}URA^{sca}_{prot}$	0.18	0.12	-0.20	-0.09	-0.23
$GUA\text{-}CYT^{sca}_{prot}$	0.35	0.55	-0.62	-0.21	-0.69
$GUA\text{-}URA^{sca}_{prot}$	0.46	0.49	-0.59	-0.31	-0.73
$GUA\text{-}ADE^{sca}_{prot}$	0.45	0.50	-0.58	-0.31	-0.72
$CYT\text{-}URA^{sca}_{prot}$	0.52	0.42	-0.53	-0.36	-0.72

1 10^{-1} 10^{-2} 10^{-3} 10^{-4} 10^{-5} 10^{-6} $<10^{-6}$

p-value

B)

	U_{mRNA}	C_{mRNA}	A_{mRNA}	G_{mRNA}	PUR_{mRNA}
$ADE\text{-}CYT^{aa}_{prot}$	-0.16	-0.53	0.39	0.29	0.56
$ADE\text{-}URA^{aa}_{prot}$	0.31	-0.14	0.03	-0.17	-0.11
$GUA\text{-}CYT^{aa}_{prot}$	0.21	-0.03	-0.53	0.41	-0.12
$GUA\text{-}URA^{aa}_{prot}$	0.31	0.22	-0.65	0.18	-0.41
$GUA\text{-}ADE^{aa}_{prot}$	0.26	0.24	-0.64	0.20	-0.38
$CYT\text{-}URA^{aa}_{prot}$	0.26	0.49	-0.39	-0.34	-0.60

1 10^{-1} 10^{-2} 10^{-3} 10^{-4} 10^{-5} 10^{-6} $<10^{-6}$

p-value

Table S7. List of all derived scales given as a Microscoft Excel document **scales.xls**: sidechain analog interaction propensity scales (SCA-energy sheet) and amino-acid interaction propensity scales (AA-energy sheet).

REFERENCES

1. Polyansky, A. A. and Zagrovic, B. (2013) Evidence of direct complementary interactions between messenger RNAs and their cognate proteins. *Nucleic Acids Res.*, **41**, 8434–8443.
