# Supplementary material for: Computational analysis of amino acids and their sidechain analogs in crowded solutions of RNA nucleobases with implications for the mRNA–protein complementarity hypothesis
Source: Nucleic Acids Res. 2014 Oct 31;42(21):12984–94. doi: 10.1093/nar/gku1035 (PMC4245939; doi:10.1093/nar/gku1035)
Supplement: SUPPLEMENTARY DATA [file supp_gku1035_nar-01911-r-2014-File009.pdf]

Sidechain analog interaction propensity scales (in kJ/mol) for naturally occurring nucleobases

	ADE	CYT	GUA	URA	ADE-CYT	ADE-URA	GUA-CYT	GUA-URA	GUA-ADE
ala	-5.95	-6.42	-0.65	-8.29	0.48	2.34	5.77	7.64	5.30
arg	63.67	-19.12	-38.58	71.14	82.79	-7.48	-19.46	-109.73	-102.25
asn	21.55	19.46	30.03	28.35	2.09	-6.79	10.57	1.68	8.47
asp	209.14	130.66	71.04	230.42	78.48	-21.28	-59.62	-159.38	-138.10
cys	-30.92	-21.09	-26.78	-28.83	-9.82	-2.08	-5.69	2.05	4.13
gln	19.05	2.40	5.92	16.74	16.65	2.31	3.52	-10.82	-13.13
glu	195.29	144.50	93.11	223.59	50.80	-28.29	-51.38	-130.47	-102.18
gly	-	-	-	-	-	-	-	-	-
his	-11.22	5.26	21.91	24.01	-16.48	-35.23	16.64	-2.10	33.13
ile	-26.50	-26.71	-36.46	-36.53	0.21	10.03	-9.75	0.07	-9.96
leu	-27.03	-26.51	-11.70	-33.20	-0.53	6.16	14.80	21.49	15.33
lys	102.70	4.87	-98.07	101.36	97.83	1.34	-102.94	-199.43	-200.77
met	-43.29	-35.13	-35.32	-42.12	-8.16	-1.17	-0.19	6.80	7.97
phe	-56.98	-47.16	-49.55	-53.71	-9.82	-3.27	-2.40	4.16	7.43
pro	-	-	-	-	-	-	-	-	-
ser	19.92	32.05	35.97	37.11	-12.13	-17.20	3.93	-1.14	16.06
thr	23.03	23.34	27.02	27.00	-0.31	-3.98	3.68	0.01	3.99
trp	-59.64	-69.26	-86.81	-72.89	9.63	13.26	-17.54	-13.92	-27.17
tyr	-46.19	-19.46	-27.26	-27.80	-26.73	-18.39	-7.80	0.54	18.93
val	-28.45	-21.36	-12.62	-25.48	-7.09	-2.96	8.74	12.87	15.83

Amino acid interaction propensity scales (in kJ/mol) for naturally occurring nucleobases

	ADE	CYT	GUA	URA	ADE-CYT	ADE-URA	GUA-CYT	GUA-URA	GUA-ADE
ala	218.71	115.65	46.72	225.72	103.06	-7.01	-68.92	-178.99	-171.99
arg	300.54	65.95	-7.55	315.08	234.59	-14.54	-73.50	-322.64	-308.09
asn	201.02	115.33	4.92	213.46	85.68	-12.44	-110.41	-208.53	-196.09
asp	395.98	300.50	114.18	405.08	95.48	-9.10	-186.32	-290.90	-281.80
cys	178.34	38.59	-32.01	165.10	139.75	13.24	-70.60	-197.12	-210.35
gln	176.67	9.04	46.25	224.21	167.63	-47.54	37.21	-177.96	-130.42
glu	414.22	245.55	128.70	462.69	168.67	-48.47	-116.85	-333.99	-285.52
gly	208.41	83.45	106.29	222.06	124.96	-13.65	22.84	-115.78	-102.12
his	149.10	63.82	-121.56	194.26	85.28	-45.15	-185.38	-315.81	-270.66
ile	189.40	79.37	-148.70	188.81	110.03	0.59	-228.06	-337.51	-338.10
leu	168.68	57.61	83.69	190.02	111.06	-21.34	26.07	-106.33	-84.99
lys	346.88	71.80	-189.52	327.61	275.08	19.27	-261.32	-517.13	-536.40
met	158.91	14.46	-2.04	163.31	144.45	-4.40	-16.50	-165.35	-160.95
phe	152.48	-4.19	-98.56	152.07	156.67	0.42	-94.38	-250.63	-251.05
pro	147.68	87.48	-123.83	179.94	60.20	-32.26	-211.31	-303.77	-271.51
ser	223.26	99.65	60.30	219.91	123.61	3.35	-39.34	-159.60	-162.96
thr	199.34	128.54	-13.76	200.11	70.80	-0.77	-142.30	-213.87	-213.10
trp	103.70	-61.63	-26.56	123.79	165.34	-20.09	35.07	-150.36	-130.27
tyr	162.17	12.56	-26.08	172.87	149.61	-10.70	-38.64	-198.94	-188.25
val	213.73	42.09	83.21	215.59	171.64	-1.86	41.12	-132.38	-130.52
